# Reduced acetylated α-tubulin in *SPAST* hereditary spastic paraplegia patient PBMCs

**DOI:** 10.3389/fnins.2023.1073516

**Published:** 2023-03-28

**Authors:** Gautam Wali, Sue-Faye Siow, Erandhi Liyanage, Kishore R. Kumar, Alan Mackay-Sim, Carolyn M. Sue

**Affiliations:** ^1^Neuroscience Research Australia and University of New South Wales, Sydney, NSW, Australia; ^2^Kolling Institute for Medical Research and Department of Medicine, University of Sydney, Sydney, NSW, Australia; ^3^Northern Clinical School, Sydney Medical School, University of Sydney, Sydney, NSW, Australia; ^4^Molecular Medicine Laboratory and Department of Neurology, Concord Repatriation General Hospital, Concord Clinical School, University of Sydney, Concord, NSW, Australia; ^5^Garvan Institute of Medical Research, Darlinghurst, NSW, Australia; ^6^Griffith Institute for Drug Discovery, Griffith University, Brisbane, QLD, Australia

**Keywords:** hereditary spastic paraplegia, microtubule, noscapine, biomarkers, neurodegenerative disease

## Abstract

HSP*-SPAST* is the most common form of hereditary spastic paraplegia (HSP), a neurodegenerative disease causing lower limb spasticity. Previous studies using HSP*-SPAST* patient-derived induced pluripotent stem cell cortical neurons have shown that patient neurons have reduced levels of acetylated α-tubulin, a form of stabilized microtubules, leading to a chain of downstream effects eventuating in increased vulnerability to axonal degeneration. Noscapine treatment rescued these downstream effects by restoring the levels of acetylated α-tubulin in patient neurons. Here we show that HSP*-SPAST* patient non-neuronal cells, peripheral blood mononuclear cells (PBMCs), also have the disease-associated effect of reduced levels of acetylated α-tubulin. Evaluation of multiple PBMC subtypes showed that patient T cell lymphocytes had reduced levels of acetylated α-tubulin. T cells make up to 80% of all PBMCs and likely contributed to the effect of reduced acetylated α-tubulin levels seen in overall PBMCs. We further showed that mouse administered orally with increasing concentrations of noscapine exhibited a dose-dependent increase of noscapine levels and acetylated α-tubulin in the brain. A similar effect of noscapine treatment is anticipated in HSP-*SPAST* patients. To measure acetylated α-tubulin levels, we used a homogeneous time resolved fluorescence technology-based assay. This assay was sensitive to noscapine-induced changes in acetylated α-tubulin levels in multiple sample types. The assay is high throughput and uses nano-molar protein concentrations, making it an ideal assay for evaluation of noscapine-induced changes in acetylated α-tubulin levels. This study shows that HSP-*SPAST* patient PBMCs exhibit disease-associated effects. This finding can help expedite the drug discovery and testing process.

## Introduction

Hereditary Spastic Paraplegia (HSP) is a neurodegenerative disorder caused by disease-causing variants in any one of at least 80 genes (Méreaux et al., 2022). HSP*-SPAST* (also known as SPG4; Lange et al., 2022) is caused by disease-causing *SPAST* variants and is the most common form of autosomal dominant HSP (59% of autosomal dominant cases; Méreaux et al., 2022). Patients with HSP*-SPAST* undergo degeneration of the corticospinal motor neurons leading to lower limb spasticity and gait disturbances. *SPAST* encodes spastin, a microtubule regulating protein (Kasher et al., 2009). We have previously evaluated the disease-mechanism of HSP*-SPAST* using patient-derived stem cell models: olfactory neurosphere-derived stem cells and induced pluripotent stem (iPS) cell derived-neurons (Abrahamsen et al., 2013; Fan et al., 2014; Wali et al., 2016, 2018, 2020). Patient-derived neuronal cells have reduced spastin levels causing reduced levels of acetylated α-tubulin, a form of stabilized microtubules (Abrahamsen et al., 2013; Wali et al., 2020). Reduced levels of acetylated α-tubulin in patient neurons leads to downstream effects of reduced microtubule-dependent axonal transport, increased oxidative stress and increased vulnerability to axonal degeneration (Wali et al., 2016, 2020). The downstream effects can be rescued by restoring acetylated α-tubulin levels in patient neurons with noscapine and other tubulin-binding drugs (Fan et al., 2014; Wali et al., 2020). In summary, our *in vitro* study findings have established that we can protect HSP*-SPAST* patient neurons from being vulnerable to degeneration by restoring acetylated α-tubulin levels.

Noscapine is an opium poppy alkaloid that does not bind to opioid receptors. It is non-narcotic, therefore non-addictive and has no effect on pain (Rida et al., 2015). It is approved in several countries as an antitussive (Rida et al., 2015). It makes a good candidate for re-purposing as a treatment for HSP*-SPAST* as it can cross the blood brain barrier and *in vitro* studies show that it rescues the downstream effects of reduced acetylated α-tubulin levels in HSP*-SPAST* patient neurons (Wali et al., 2020). At present, it is not known if orally administered noscapine can increase the cellular content of acetylated α-tubulin in the brain *in vivo*.

Identifying disease-associated effects in the blood can help expedite the drug discovery and testing process. Microtubule expression is not restricted to neuronal cells that are degenerated in HSP*-SPAST* patients. Based on this, we hypothesized that acetylated α-tubulin levels are also reduced in non-neuronal patient cells. The first aim of this study was to compare the expression of acetylated α-tubulin between HSP*-SPAST* patients and healthy controls in peripheral blood mononuclear cells (PBMCs).

The second aim of this study was to test if noscapine can cross the blood–brain barrier and increase the content of acetylated α-tubulin in the brain. For this, we orally administered mouse with increasing doses of noscapine and measured the levels of noscapine and acetylated α-tubulin in the mouse brain. Acetylated α-tubulin was measured in mouse brain homogenate protein samples using the highly sensitive Homogeneous Time Resolved Fluorescence (HTRF) assay technology (Degorce et al., 2009). Human brain cells are relatively large (cortical neurons extend up to 1 m in length) and subsequently have higher expression of microtubules compared to smaller cell types especially PBMCs (that are about 50–100 μm long). The third aim of the study was to test if the HTRF assay can detect acetylated α-tubulin expression and noscapine induced changes in acetylated α-tubulin expression in human PBMCs. This assay will be relevant to future studies testing noscapine drug treatment effects on acetylated α-tubulin expression in non-neuronal cells for HSP-*SPAST* and other diseases.

Overall, we investigated if HSP*-SPAST* patient PBMCs reflect the disease associated pathological effects of reduced acetylated α-tubulin seen in neuronal cells, and if noscapine can cross the blood brain barrier to increase the levels of acetylated α-tubulin in the brain. Finally, we investigate HTRF as a highly sensitive high throughput assay to measure the effects of noscapine treatment in mouse brain (*in vivo*) and human PBMCs (*in vitro*).

## Methods

### Human ethics compliance and patient recruitment

Experiments involving human PBMCs were approved by the Northern Sydney Local Health District Human Research Ethics Committee, Australia (Reference number: RESP/15/314) and written informed consent was obtained from all participants. Twenty participants, i.e., 9 *SPAST* patients and 11 healthy controls were recruited to this study from the Neurogenetics Clinic, Royal North Shore Hospital, Sydney, Australia. The patients in this study were examined by movement disorder specialists (CS, SFS, and KRK) and had a confirmed diagnosis of HSP*-SPAST* on genetic testing. Disease-severity of the patients was measured using the HSP clinical rating scale, Spastic Paraplegia Rating Scale (SPRS; Schüle et al., 2006). Details of the study participants are presented in Table 1.

**Table 1:** Details of individuals with HSP-*SPAST* (*SPG4*) and healthy controls.

Sample	Gender	Age	Diagnosis	SPRS	*SPAST* variant (NM_014946.4)
**Healthy controls**					
19/009	F	58	Control		
19/048	M	34	Control		
19/056	M	33	Control		
12/013	F	47	Control		
19/007	F	34	Control		
19/052	F	56	Control		
19/059	F	22	Control		
19/058	M	37	Control		
19/014	F	31	Control		
19/054	F	31	Control		
19/053	F	40	Control		
**Individuals with HSP-*SPAST* (SPG4)**					
08/088	M	81	HSP*-SPAST*	22	c.444G>A (p.Trp148Ter)
06/042	F	58	HSP*-SPAST*	49	c.1392A>T (p.Glu464Asp)*
09/010	F	53	HSP*-SPAST*	10	c.1291C>T (p.Arg431Ter)
18/268	M	58	HSP*-SPAST*	23	c.1413+1G>T
09/017	F	73	HSP*-SPAST*	24	Duplication of exon 16*
04/011	M	73	HSP*-SPAST*	22	c.1196C>T (p.Ser399Leu)*
17/114	F	36	HSP*-SPAST*	2	c.451_454del (p.Lys151fs)
14/074	F	51	HSP*-SPAST*	1	c.1291C>T (p.Arg431Ter)
09/016	M	46	HSP*-SPAST*	20	Duplication of exon 16*.

SPRS, Spastic paraplegia Rating Scale. *(Vandebona et al., 2012).

### Blood sample collection, PBMC isolation, and PBMC acetylated α-tubulin measurement using flow cytometry

#### Blood collection

Fifty milli liter (mL) of peripheral blood was collected for each participant in 5 × 10 mL heparin tubes. Samples were processed for PBMCs within 4 h of collection.

#### PBMC isolation

PBMCs were isolated from whole blood collected in 10 mL heparin blood tubes using Ficoll–Hypaque density gradient (Fuss et al., 2009). For each 10 mL blood sample, blood sample was diluted with Phosphate-buffered saline (PBS) + 2% Fetal Bovine Serum (1:1 volume ratio) to form a 20 mL diluted sample solution. 15 mL volume of Ficoll–Hypaque density gradient solution was added to a 50 mL falcon tube. The diluted sample was layered on top of the Ficoll–Hypaque solution. The tube was centrifuged at 900 g for 20 min with the brakes off. The upper plasma layer was removed and the mononuclear cells at the interphase were harvested (the layer above the Ficoll–Hypaque solution). Mononuclear cells were washed twice in PBS buffer +2% Fetal Bovine Serum solution. Finally, PBMCs were counted and frozen down in CryoStor® CS10 freezing media (Catalog# 07930, Stem cell technologies) and stored in cryo tanks for long term storage.

#### PBMC immunostaining

Frozen PBMCs were thawed in RPMI cell culture media: RPMI media (Catalog# 11875-093, Thermo Fisher scientific) with 10% FBS. To identify viable cells, PBMCs were incubated with (Live/Dead Fixable Near IR Viability Dye, Catalog# L34975 Thermo Fisher Scientific) at a concentration of 1:1,000 for 15 min. The cells were then washed twice with PBS. To identify PBMC cell sub-types, PBMCs were incubated with a cocktail of surface antibodies—CD14 (1:20, Catalog# 301830, BioLegend), CD2 (1:20, Catalog# 309208 BioLegend) and CD19 (1:20, Catalog# 302212, BioLegend) for 30 min. The cells were then washed twice with PBS. PBMCs were fixed and permeabilised using the Cytofix/Cytoperm Fixation/Permeabilization solution kit (Catalog# 554714, BD Biosciences), as described before (Wali et al., 2021). PBMCs were fixed using the CytoFix reagent for 25 min, followed by two CytoPerm reagent washes. PBMCs were permeabilised using the CytoPerm reagent for 30 min. PBMCs were immunostained by incubating PBMCs with the conjugated anti-acetylated α tubulin antibody at a dilution of 1:50 for 30 min [(Bonser et al., 2021), Catalog# sc-23950, Santacruz], followed by two CytoPerm reagent washes. After immunostaining, PBMCs were resuspended in 500 μL PBS and filtered through a 70 μm cell strainer.

#### Flow cytometry and data analysis

**Figure 1:**
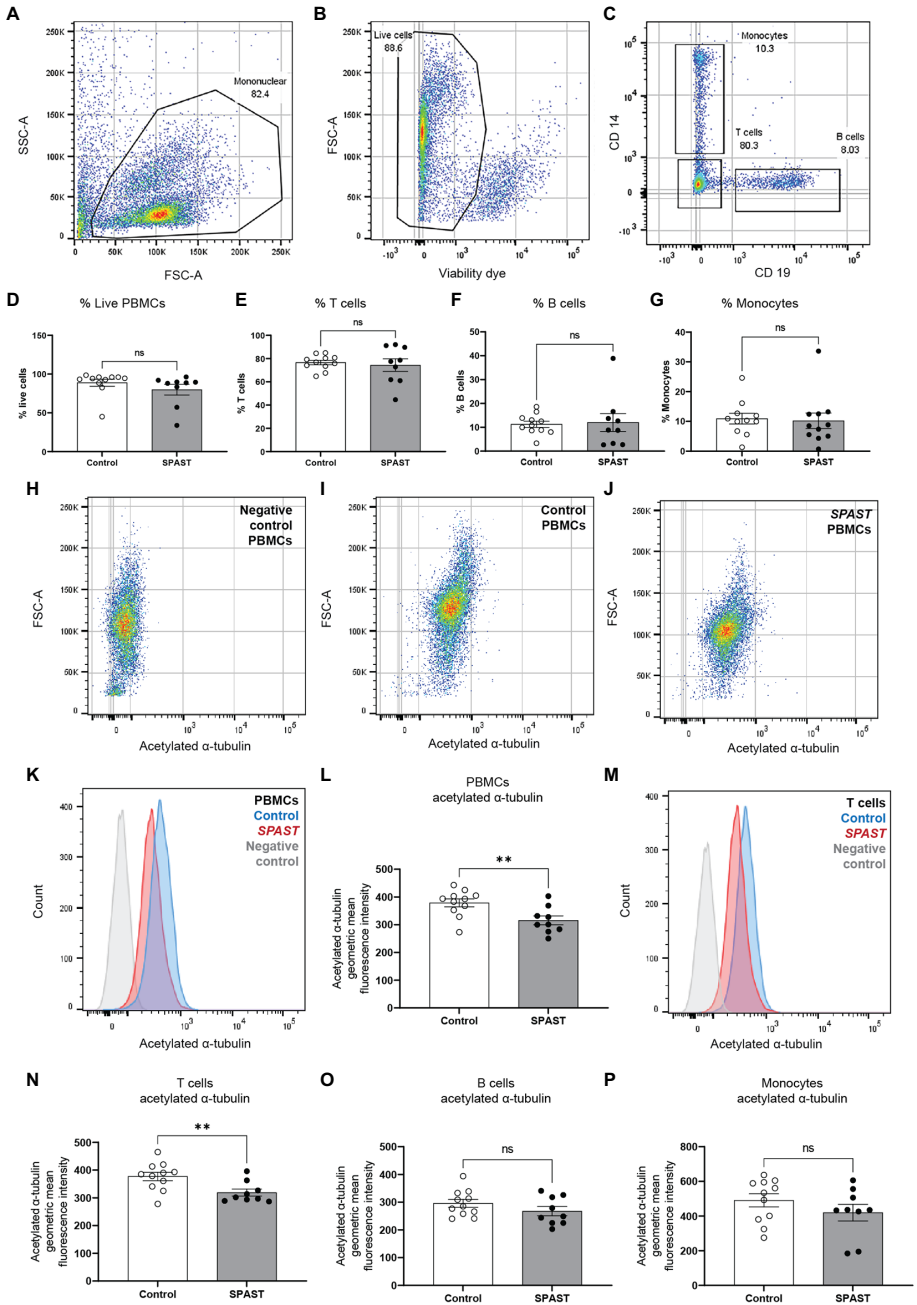
Acetylated α-tubulin levels in *SPAST* HSP patient and control PBMCs. Flow cytometry was used to measure acetylated α-tubulin in overall PBMCs and PBMC cell sub-types. **(A)** The forward-scatter and side-scatter (FSC/SSC) gated plots represent the total mononuclear cells. **(B)** Live PBMCs were identified using a cell viability dye. Viable cells have a relatively lower fluorescence level of the viability dye and vice versa for non-viable cells. Gating was applied to identify the proportion of viable cells. **(C)** CD2, CD19, and CD14 cell surface antibodies were used to identify PBMC cell sub-types T cells, B cells and monocytes. The scatter plot shows CD14 monocytes and CD19 B cells separated using the surface antibodies. The proportion of live PBMCs **(D)** and the proportion of T cells **(E)**, B cells **(F)** and monocytes **(G)** is shown for all HSP*-SPAST* patients and healthy controls. **(H–J)** Levels of acetylated α-tubulin in PBMCs were measured in patient and control PBMC samples. Scatter plot of an acetylated α-tubulin negative control sample **(H)** and representative control **(I)** and patient **(J)** samples are shown. **(K)** Histogram plot showing acetylated α-tubulin levels in representative patient and control PBMC samples. **(L)** Patient and control group level comparison based on acetylated α-tubulin geometric mean fluorescence intensity in all PBMCs. **(M)** Histogram plot showing acetylated α-tubulin levels in representative patient and control T cells. Patient and control group level comparison of T cells **(N)**, B cells **(O)** and monocytes **(P)** based acetylated α-tubulin geometric mean fluorescence intensity. Mean ± SEM.

The PBMC sample fluorescence levels were measured using the BD LSRFortessa™ flow cytometer. Data analysis was performed using FlowJo™, a flow cytometry data analysis software. Compensation controls for PE stained anti-CD2 antibody, APC stained anti-CD19 antibody, BV421 stained anti-CD14 antibody and AF488 stained anti-acetylated α-tubulin antibody were used to adjust for fluorophore spill over. Fluorescence minus one (FMO) controls for Near IR and AF488 were used to gate for viable cells and set the threshold for positive acetylated α-tubulin readings (Maecker and Trotter, 2006). FMO control for AF488 is presented in Figures 1H,K,M as the negative control for acetylated α-tubulin. Acetylated α-tubulin fluorescence was measured in all viable PBMCs, CD2 positive T cells, CD19 positive B cells, and CD14 positive monocytes for each sample. The acetylated α-tubulin geometric mean fluorescence intensity was measured from individual samples for group level comparisons.

### Animal ethics compliance

Study involving mouse was conducted in accordance with the guidelines set out in the National Health and Medical Research Council, Australian Code of Practice for the Care and Use of Animals for Scientific Purposes, 8th edition, 2013 (1). The study was assessed and approved by the University of Queensland Animal Ethics Committee and assigned the following project code: TETRAQ/330/19/388/18/M ‘Noscapine HCL (Noscapine): Tissue Distribution and Pharmacokinetic Analysis of Novel Compounds in Rodents’.

### Mouse noscapine treatment, blood and brain sample preparation and mouse brain protein extraction

#### Mouse type

C57BL/6 J mouse are an acceptable and commonly used strain and species for pharmacokinetic and pharmacodynamic studies. The mice used were 7–8 weeks old and they weighed 20.83–23.13 g. Mouse were subject to a 5-day acclimation period prior to noscapine administration. Only animals without visible signs of illness were used for the study.

#### Animal housing conditions

Animals were housed in their study groups with up to four animals per individually ventilated cage (IVC). The IVC units had a minimum of 15 air changes per hour. Environmental enrichment was added to each IVC. These items included chewsticks, rodent hutches and nesting material. The animal housing temperature was 22.5°C ± 2.5°C and humidity of 50% ± 20%. The animal house had a 12-h light/dark cycle.

#### Study design

The study design is summarized in Table 2. On completion of the acclimation period, mouse were administered with noscapine (3 groups, *n* = 4 mouse per group, Table 2). Noscapine treatment was administered as a bolus oral dose by gavage in a volume of 10 mL/kg.

**Table 2:** Mouse noscapine treatment study plan.

Study group	Noscapine dose (mg/kg)	Route	C57BL/6 J mouse	Terminal samples collection time points
1	100	Oral	4	60 min post-dose
2	200	Oral	4	60 min post-dose
3	400	Oral	4	60 min post-dose

#### Mouse plasma processing

Blood samples were collected via the inferior vena cava and transferred into tubes containing K3-EDTA which had been previously cooled on ice. Whole blood was transferred to a separate tube and red blood cells lysed using Red Blood Cell Lysis Buffer (ab204733, Abcam) to collect plasma. The processed plasma was stored at −80°C.

#### Mouse brain tissue processing

Mouse were euthanised by an overdose of pentobarbitone (administered via intraperitoneal injection). Immediately following euthanasia, the entire brain was collected and homogenized in ice-cold phosphate buffered saline containing 1× protease inhibitor cocktail (Sigma-Aldrich, Catalog# P8340). The brain tissue was homogenized using the Retsch mixer mill mm 400 homogenizer and two stainless steel grinding balls (3 mm, Retsch) using these conditions: 10 Hz for 2 min at 4°C + 30 Hz for 7 min at 4°C. The resulting homogenized brain sample was stored at −80°C.

#### Mouse brain homogenate protein extraction

One hundred micro liter of the brain homogenate was mixed with 300 μL RIPA lysis buffer (Sigma, Catalog# R0278-50ML) supplemented with 1x Protease Inhibitor Cocktail (Thermo Fisher scientific, Catalog# 78429). The sample mixture was mixed by pipetting and vortexing. The sample mixture was kept on ice and agitated on a shaker for 2 h. The sample mixture was centrifuged for 20 min at 4°C at 16,000 rcf. Supernatant containing the protein was collected and protein concentration was measured using the BCA protein estimation kit (Thermo Fisher scientific, Catalog# 23225).

### Measuring noscapine concentrations in mouse plasma and brain using liquid chromatography tandem mass spectrometry

Twenty micro liter (µL) aliquots of mouse plasma and brain homogenate were spiked with 1-Hydroxymidazolam internal standard, and the samples were then analyzed using the liquid chromatography tandem mass spectrometry (LC-MS/MS) method. Plasma and brain homogenate concentrations of noscapine were back calculated from calibration curves. The lower limit of quantification (LLOQ) for this method was 1 ng/mL. The LC-MS/MS chromatograms of plasma and brain homogenate with and without noscapine (150 ng/mL) and internal standard 1-Hydroxymidazolam (1 μg/mL) are shown in Supplementary Figures 2, 3.

### *In vitro* PBMCs noscapine treatment and PBMC protein extraction

The human PBMCs used to test *in-vitro* effects of noscapine treatment (*n* = 8 PBMC cell lines) were purchased from Stemcell technologies (Catalog#: 70025.1). The PBMCs were cultured in RPMI cell culture media: RPMI media (Catalog# 11875-093, Thermo Fisher scientific) with 10% FBS at 37°C with 5% CO_2_. The PBMCs were treated with 10 μM noscapine (Cayman chemical, Catalog# 17255) for 1 h.

#### Human PBMC protein extraction

PBMCs cultured in RPMI media were transferred to a 15 mL falcon tube and centrifuged at 500 g for 10 min. PBMC cell pellet was resuspended in 50 μL of cell lysis reagent (Sigma, Catalog# C3228) supplemented with 1x Protease Inhibitor Cocktail (Thermo Fisher scientific, Catalog# 78429). The sample mixture was mixed by pipetting and vortexing. Then, the sample was kept on ice and agitated on a shaker for 20 min. The sample was then centrifuged at 4°C at 16,000 rcf. Supernatant containing the protein was collected and protein concentration was measured using the BCA protein estimation kit (Thermo Fisher scientific, Catalog# 23225).

### Acetylated α-tubulin measurement in mouse brain and human PBMCs using the HTRF acetylated α-tubulin assay

We used the HTRF Alpha-Tubulin acetyl-K40 kit (Perkin Elmer, Catalog# 63ADK072PEG) to measure the relative levels of acetylated α-tubulin. In this study, we have measured the levels of acetylated α-tubulin in the mouse brain and human PBMCs. The expression levels of acetylated α-tubulin can vary between different tissue types. A serial protein dilution was used to identify the optimal protein concentration required to measure acetylated α-tubulin for each tissue sample type. For both mouse brain and human PBMC samples, a dilution series was prepared for the protein samples in pure water: 10, 5, 1, 0.1, 0.05, 0.01, and 0.001 ng/16 μL. 4 μL of the antibody mixture, i.e., 2 μL of d2-antibody solution and 2 μL of Cryptate-antibody solution was added to each protein dilution solution (both the antibodies bind to acetylated α-tubulin and subsequently generating Fluorescence resonance energy transfer (FRET)). The total reaction volume per sample is 20 μL. The assay was performed in a 384 well plate. Each well can hold 20 μL sample. The sample solution was incubated for 2 h at room temperature. The fluorescence was measured using the HTRF compatible fluorescence plate reader (Nivo, Perkin Elmer). 5 and 0.01 ng/μL were identified to be the optimal protein concentration to measure acetylated α-tubulin in human PBMCs and mouse brain samples, respectively. The average time required for this assay from protein sample to data analysis is 4 h.

### Statistical analysis

Prism (GraphPad) was used to analyze data and plot graphs. Analysis of data presented was performed using one-way analysis of variance with Tukey’s multiple comparisons test, Student’s *t*-test and Person’s correlation coefficient analysis.

## Results

### HSP*-SPAST* patient PBMCs have reduced acetylated α-tubulin

To test if non-neuronal HSP-*SPAST* patient cells have disease-associated pathology of reduced acetylated α-tubulin, we compared the expression of acetylated α-tubulin in PBMCs from 9 HSP-*SPAST* patients and 11 healthy control PBMCs. Acetylated α-tubulin levels were measured using flow cytometry in overall PBMCs (Figures 1A,B) and in PBMC cell sub-types, i.e., CD2 positive T-cell lymphocytes, CD19 positive B-cell lymphocytes and CD14 positive monocytes (Figure 1C).

First, we tested the proportion of PBMC cell sub-types between the patient and control groups. There was no difference in the proportion of PBMC cell sub-types between the HSP-*SPAST* patients and healthy controls. Both patient and control groups had about 90% live PBMCs (Figure 1D). Of these live PBMCs, about 80% of PBMCs were T-cells (Figure 1E), 10% were B-cells (Figure 1F) and 10% were monocytes (Figure 1G). The proportion of the different PBMC cell subtypes was as expected and not affected by disease status.

Figures 1H–J show acetylated α-tubulin expression dot plots of a representative negative control (Figure 1H), healthy control PBMC sample (Figure 1I) and patient PBMC sample (Figure 1J). The same data is presented as a histogram in Figure 1K. Acetylated α-tubulin levels in the HSP-*SPAST* patient PBMCs was significantly lower than control PBMCs (Figures 1L, *p* < 0.01). Assessment of acetylated α-tubulin levels in the PBMC cell sub-types showed that patient T-cells had significantly lower acetylated α-tubulin levels compared to control T-cells (Figures 1M,N). Patient B cells (Figure 1O) and monocytes (Figure 1P) showed a trend of reduced acetylated α-tubulin levels, but this effect was not statistically significant.

The average age of the 9 HSP*-SPAST* patients (4 males and 5 females) and 11 healthy controls (3 males and 8 females) is 58.78 and 38.45 years, respectively. Correlation analysis did not identify any correlation between the age of the control and patient participants vs. their levels of acetylated α-tubulin (Supplementary Figure 1; controls: R^2^ = 0.03 and HSP*-SPAST*: R^2^ = 0.22).

Correlation analysis did not identify a significant correlation between the SPRS and acetylated α-tubulin levels in HSP-*SPAST* patients (R^2^: 0.041).

### Orally administered noscapine was detected in mouse brain

**Figure 2:**
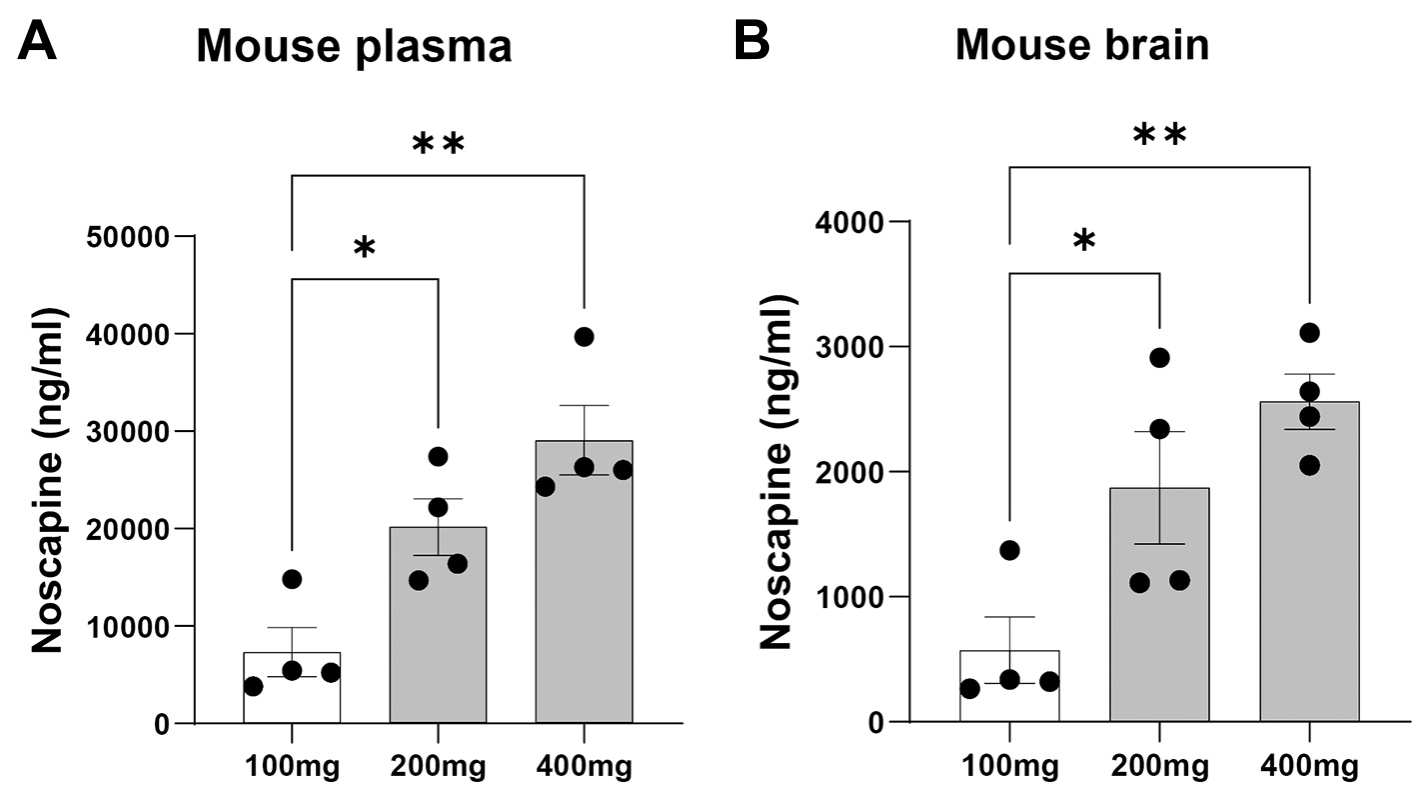
Plasma and brain noscapine levels in noscapine treated mouse. Mouse was orally administered with increasing concentrations of noscapine: 100, 200, and 400 mg/kg. Noscapine was measured in mouse plasma **(A)** and brain homogenate **(B)** at 60 min post-dose using Liquid Chromatography Tandem Mass Spectrometry (LC-MS/MS). *N* = 4 mouse per group. One way ANOVA confirmed a significant noscapine treatment effect in the mouse plasma (*p* < 0.01) and brain (*p* < 0.01). Tukey’s *post-hoc* multiple comparisons showed group differences (^*^p < 0.05 and ^**^p < 0.01). Mean ± SEM.

To test if orally administered noscapine can cross the blood brain barrier, three groups of mouse (*n* = 4 mouse per group) were orally administered with increasing concentrations of noscapine, i.e., 100, 200, and 400 mg/kg. No morbidity or mortality was observed during the acclimation and the study period. Body weight was recorded once during the acclimation period and on the day of dosing. No changes in body weight were observed post-dosage. Body weight (g) ± SD of noscapine treated mouse: 100 mg/kg—22.39 ± 1.07, 200 mg/kg mouse—22.05 ± 0.92, and 400 mg/kg mouse—21.83 ± 0.87. Noscapine was measured using Liquid Chromatography with tandem mass spectrometry (LC-MS/MS) in the mouse plasma and brain following oral administration of noscapine at 60 min post-dose. Noscapine treatment increased the levels of noscapine in the mouse plasma and brain (Figures 2A,B). ANOVA indicated a significant effect of noscapine treatment in the mouse plasma (Figure 2A, *p* < 0.01) and brain (Figure 2B, *p* < 0.01). Tukey’s *post-hoc* multiple comparisons of both mouse plasma and brain noscapine levels indicated that the noscapine levels in 100 mg/kg noscapine treated mouse was significantly different from 200 mg/kg noscapine treated mouse (Figures 2A,B, *p* < 0.05) and 400 mg/kg noscapine treated mouse (Figures 2A,B *p* < 0.01). Noscapine treatment showed a tissue dependent affect. Comparison of noscapine levels in mouse plasma and brain showed that noscapine concentration in the brain is about 12-fold lower than in plasma (for example, at 100 mg/kg dosage, brain noscapine average: 573 ng/mL vs. plasma noscapine average: 7327 ng/mL). There was a significant correlation between noscapine levels in plasma and brain samples. Spearman correlation coefficient analysis showed a significant correlation in noscapine levels in plasma and brain samples for the 100 and 200 mg/kg treatment groups (Table 3).

**Table 3:** Correlation of noscapine levels in plasma and brain of noscapine treated mouse.

Noscapine treatment in mouse	Pearson correlation coefficient	
	R squared	*p*-Value
100 mg/kg	0.969	0.015
200 mg/kg	0.97	0.015
400 mg/kg	0.675	0.177

### Noscapine dose-dependent increase of acetylated α-tubulin levels

**Figure 3:**
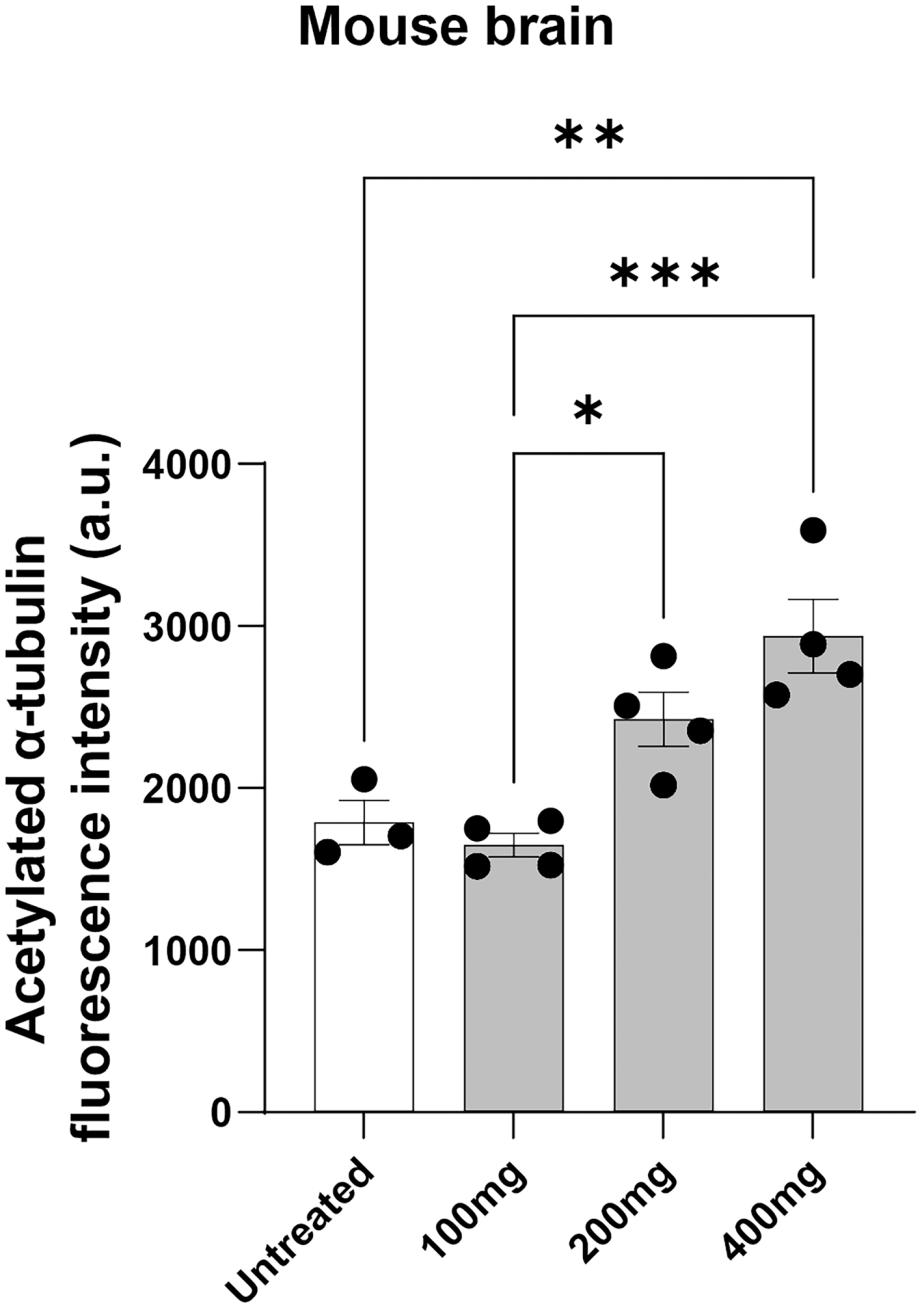
Acetylated α-tubulin levels in noscapine treated mouse. Acetylated α-tubulin was measured in the brain of the mouse dosed with noscapine. Mouse was orally administered with increasing concentrations of noscapine: 100, 200, and 400 mg/kg. One way ANOVA confirmed a significant noscapine treatment effect on acetylated α-tubulin in the mouse brain (*p* < 0.01). Tukey’s *post-hoc* multiple comparisons showed group differences (^*^p < 0.05, ^**^p < 0.01, ^***^p < 0.001). *N* = 3 or 4 mouse per group. a.u. = arbitrary unit. Mean ± SEM.

Acetylated α-tubulin was measured in homogenized brain lysates using the highly sensitive HTRF Alpha-Tubulin acetyl-K40 assay. Brain samples from mouse treated with noscapine showed a dose-dependent increase in the levels of acetylated α-tubulin (Figure 3). ANOVA indicated a significant effect of noscapine treatment on the levels of acetylated α-tubulin in the mouse brain (*p* < 0.001). Tukey’s *post-hoc* multiple comparisons of the mouse brain acetylated α-tubulin levels indicate that the acetylated α-tubulin in the untreated mouse was significantly different from mouse treated with 400 mg/kg noscapine (*p* < 0.01; Figure 3). The level of acetylated α-tubulin in 100 mg/kg noscapine treated mouse was significantly different from mouse treated with 200 mg/kg (*p* < 0.05) and 400 mg/kg (*p* < 0.001). These results show that orally administered noscapine can increase the levels of acetylated α-tubulin in the mouse brain. This effect can be achieved at a noscapine dosage of 400 mg/kg or higher.

### HTRF assay can detect noscapine-induced acetylated α-tubulin changes in human PBMCs

**Figure 4:**
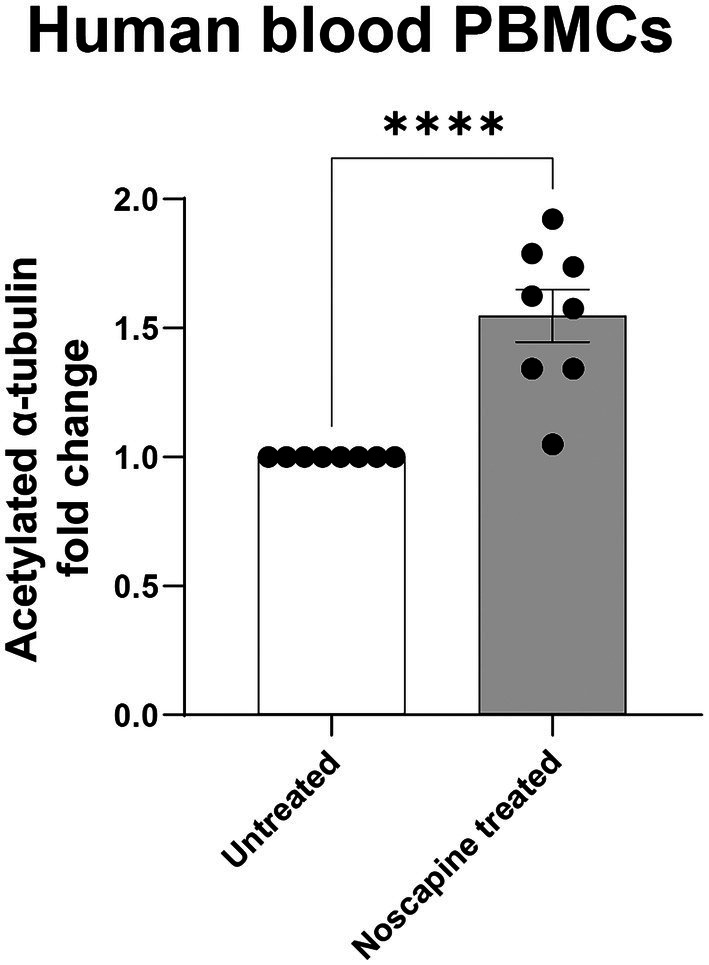
Acetylated α-tubulin levels in noscapine treated human PBMCs. Acetylated α-tubulin was measured in human PBMCs treated with noscapine (*in vitro*) at 10 μM for 1 h. Student’s t-test confirmed a significant noscapine treatment effect on acetylated α-tubulin levels (^****^p < 0.0001). *N* = 8 PBMC samples per untreated and noscapine treated groups. Mean ± SEM.

To test if the HTRF Alpha-Tubulin acetyl-K40 assay can detect changes in acetylated α-tubulin levels in human PBMCs, we treated PBMCs with 10 μM noscapine *in-vitro* for 1 h. Acetylated α-tubulin levels in the noscapine treated PBMCs was 1.5 fold higher than untreated PBMCs (*p* < 0.0001; Figure 4).

## Discussion

In this study, we show that acetylated α-tubulin level is reduced in HSP*-SPAST* patient PBMCs compared to healthy control PBMCs. This finding supports our hypothesis that non-neuronal cells, PBMCs, from patients with HSP*-SPAST* show the disease-associated effect of reduced acetylated α-tubulin, similar to previous findings in patient-derived neuronal cells (Abrahamsen et al., 2013; Wali et al., 2020). We investigated acetylated α-tubulin levels in multiple PBMC subtypes, i.e., T and B cell lymphocytes and monocytes to understand how acetylated α-tubulin levels differed in each subtype. The levels of acetylated α-tubulin in patient T cell lymphocytes were significantly lower compared to control T cell lymphocytes. But the levels of acetylated α-tubulin were comparable between patient and control B cell lymphocytes and monocytes. It is likely that the high proportion of T cells in the PBMCs (80% of all PBMCs) contributed to the patient vs. control differences seen in the overall PBMCs.

The expression of microtubules is not restricted to neuronal cells, that are degenerated in HSP-*SPAST* patients, but are also expressed in non-neuronal cell types. Therefore, it is plausible for the HSP-*SPAST* disease associated effect of reduced acetylated α-tubulin to be present in non-neuronal cell types. Our results show that PBMCs, an easily accessible source of cells exhibit HSP-*SPAST* disease-associated effects, thus showing that HSP-*SPAST* PBMCs can be used to study disease pathology and potentially develop a biomarker for the disease. To our knowledge, this is the first study showing HSP disease-associated effects in PBMCs. Similar to our findings, disease-associated effects have been shown in PBMCs for multiple neurodegenerative diseases. For example, evaluation of PBMCs from patients with Parkinson’s disease and healthy controls showed that patient PBMCs have increased levels of mitochondrial reactive oxygen species and reduced levels of the antioxidant superoxide dismutase (Smith et al., 2018). Other blood components have been used to study disease-associated effects in HSP. Serum neurofilament light chain (sNfL) is an indicator of neuronal damage. Evaluation of sNfL levels in 93 patients with HSP-*SPAST* and 60 controls showed that sNfL levels was increased in the patients (Wilke et al., 2018). However, increased sNfL has been observed in multiple neurodegenerative disorders including motor neuron disease (Nardo et al., 2011) and multiple sclerosis (Benkert et al., 2022) and is not HSP*-SPAST* disease specific.

As described in the introduction, we have previously evaluated the disease-mechanism and identified a possible drug treatment candidate for HSP-*SPAST* using patient-derived stem cell models: olfactory neurosphere-derived stem cells and iPS cell derived-neurons (Abrahamsen et al., 2013; Fan et al., 2014; Wali et al., 2016, 2018, 2020). We have shown that tubulin-binding drug noscapine can increase the levels of acetylated α-tubulin in patient neurons and rescue their vulnerability to degeneration. The second aim of the study was to test if orally administered noscapine can cross the blood brain barrier and increase the level of acetylated α-tubulin in the brain. Previous studies have shown that when taken orally, noscapine can cross the blood brain barrier (Rahmanian-Devin et al., 2021). In humans, mean plasma concentration after a single 150 mg oral dose reaches a peak at 1 h (Dahlström et al., 1982). Similar kinetics were observed after 100, 200, and 300 mg doses (Karlsson et al., 1990). The terminal half-life in humans was estimated at 4.5 h or longer and bioavailability calculated to be 30% with 3.6-fold inter-individual variation (Dahlström et al., 1982; Karlsson et al., 1990). Similar to humans, in mouse administered with 75, 150, and 300 mg/kg oral noscapine, the mean plasma noscapine concentrations peaked at 1.12, 1.50, and 0.46 h, respectively (Aneja et al., 2007). The mean percent oral bioavailability of noscapine was 22.76%, 45.05%, and 26.6% at 75, 150, and 300 mg/kg, respectively (Aneja et al., 2007). In our experiments, we dosed adult mouse by gavage with 100, 200, 400 mg/kg of noscapine. At 1 h after oral gavage there was a dose-dependent increase in noscapine levels in the mouse plasma and brain as well as a dose-dependent increase in the levels of acetylated α-tubulin in mouse brain. Our study provides evidence that orally administered noscapine can increase the levels of acetylated α-tubulin in the brain. This *in vivo* result supports the use of noscapine as a potential therapeutic agent for HSP*-SPAST*.

Noscapine-induced changes in acetylated α-tubulin levels in the mouse brain (*in vivo* treatment) and human PBMCs (*in vitro* treatment) were detected using a HTRF based assay. This assay is shown to be highly sensitive and most importantly can be miniaturized into the 384 well plate format (Degorce et al., 2009). The HTRF assay uses nanogram protein concentrations and the total reaction volume per sample is 20 μL. The low protein concentration required and the ability to perform this assay in a 384 well plate makes this assay ideal for evaluation of acetylated α-tubulin in large batches of samples, for example clinical trials where multiple samples collected at multiple different timepoints can be analyzed together avoiding any batch-to-batch effects. Therefore, we propose this assay as a sensitive high-throughput assay to measure the effect of noscapine treatment.

One of the limitations of this study is the lack of appropriately age and gender matched healthy controls. The recruitment of study participants for this study was affected by the COVID-19 global pandemic. However, correlation analysis did not identify any correlation between the age of the study participants vs. their levels of acetylated α-tubulin, suggesting that the reduced levels of acetylated α-tubulin levels in the patient PBMCs is a disease-associated effect. Evaluation of a larger cohort with more appropriately age matched controls and disease-controls, particularly including mutations associated with microtubule function (for example: KIF1A/SPG30, ATL1/SPG3A) are required to establish the reduced levels of acetylated α-tubulin in PBMCs as a HSP-*SPAST* specific disease biomarker.

In summary, we show that (a) easily accessible patient PBMCs show HSP*-SPAST* disease-associated pathology of reduced levels of acetylated α-tubulin (b) orally administered noscapine can increase levels of acetylated α-tubulin in mouse brain and (c) the HTRF acetylated α-tubulin assay can detect noscapine induced changes in acetylated α-tubulin levels in multiple tissue types. Therefore, we propose measurement of acetylated α-tubulin levels using HTRF to quantify the treatment effects of noscapine in patients with HSP*-SPAST* at the cellular level.

## Data availability statement

The original contributions presented in the study are included in the article/Supplementary material, further inquiries can be directed to the corresponding author.

## Ethics statement

The experiments involving human PBMCs was approved by the Northern Sydney Local Health District Human Research Ethics Committee, Australia (Reference number: RESP/15/314) and written informed consent was obtained from all participants. The patients/participants provided their written informed consent to participate in this study. Experiments involving mice were assessed and approved by the University of Queensland Animal Ethics Committee and assigned the following project code: TETRAQ/330/19/388/18/M ‘Noscapine HCL (Noscapine): Tissue Distribution and Pharmacokinetic Analysis of Novel Compounds in Rodents’.

## Author contributions

GW, AM-S, and CS designed the study. S-FS, KK, and CS recruited patients to the study. S-FS, GW, and EL performed the experiments measuring acetylated α-tubulin. GW performed data analysis. GW, AM-S, and CS provided funding. CS provided facilities for the experiments. AM-S and GW wrote and edited the first draft. All authors contributed to the article and approved the submitted version.

## Funding

We are most grateful to the Hereditary Spastic Research Foundation Inc. and the Spastic Paraplegia Foundation Inc. for funding the study.

## Conflict of interest

The authors declare that the research was conducted in the absence of any commercial or financial relationships that could be construed as a potential conflict of interest.

## Publisher’s note

All claims expressed in this article are solely those of the authors and do not necessarily represent those of their affiliated organizations, or those of the publisher, the editors and the reviewers. Any product that may be evaluated in this article, or claim that may be made by its manufacturer, is not guaranteed or endorsed by the publisher.

## Supplementary material

Supplementary Figure 1 Correlation of control and HSP-*SPAST* patient acetylated α-tubulin levels to age. Acetylated α-tubulin levels in controls **(A)** and HSP-*SPAST* patient **(B)** PBMCs were correlated to their age. Pearson correlation coefficient showed no age-related correlation with acetylated α-tubulin levels in both control (R^2^: 0.03) and patient groups (R^2^: 0.22).

Supplementary Figure 2 Chromatograms of noscapine treated mouse plasma. Chromatograms of noscapine and 1-Hydroxymidazolam measured in **(A, B)** blank plasma, **(C, D)** plasma spiked with 1-Hydroxymidazolam (internal standard, 1µg/ml) and **(E, F)** plasma spiked with noscapine (150ng/ml) and 1-Hydroxymidazolam (internal standard, 1µg/ml).

Supplementary Figure 3 Chromatograms of noscapine treated mouse brain homogenate. Chromatograms of noscapine and 1-Hydroxymidazolam measured in **(A, B)** blank brain homogenate, **(C, D)** brain homogenate spiked with 1-Hydroxymidazolam (internal standard, 1µg/ml) and **(E, F)** brain homogenate spiked with noscapine (150ng/ml) and 1-Hydroxymidazolam (internal standard, 1µg/ml).
