# Supplementary figures and images for: Reduced acetylated α-tubulin in SPAST hereditary spastic paraplegia patient PBMCs
Source: Front Neurosci. 2023 Mar 28;17:1073516. doi: 10.3389/fnins.2023.1073516 (PMC10152469; doi:10.3389/fnins.2023.1073516)

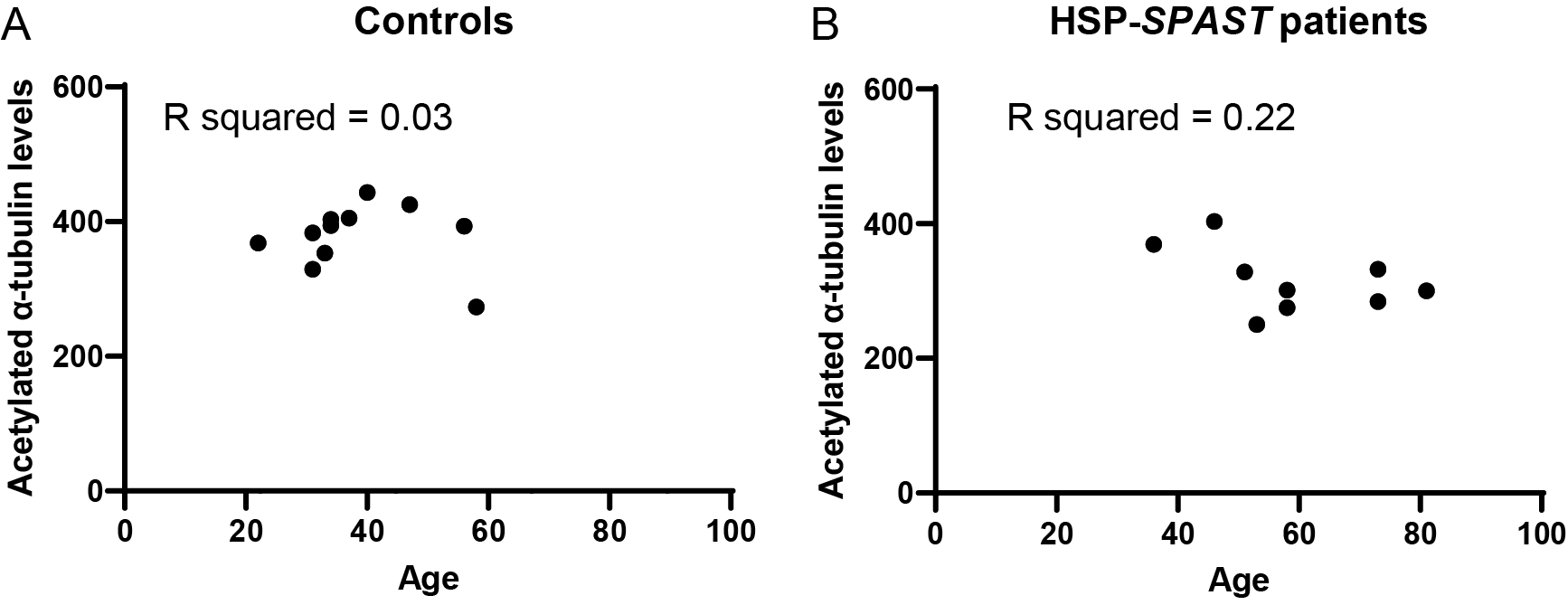

Supplement: Supplementary Figure 1 — Correlation of control and HSP-SPAST patient acetylated α-tubulin levels to age. Acetylated α-tubulin levels in controls (A) and HSP-SPAST patient (B) PBMCs were correlated to their age. Pearson correlation coefficient showed no age-related correlation with acetylated α-tubulin levels in both control (R2: 0.03) and patient groups (R2: 0.22). [file Image_1.tif]

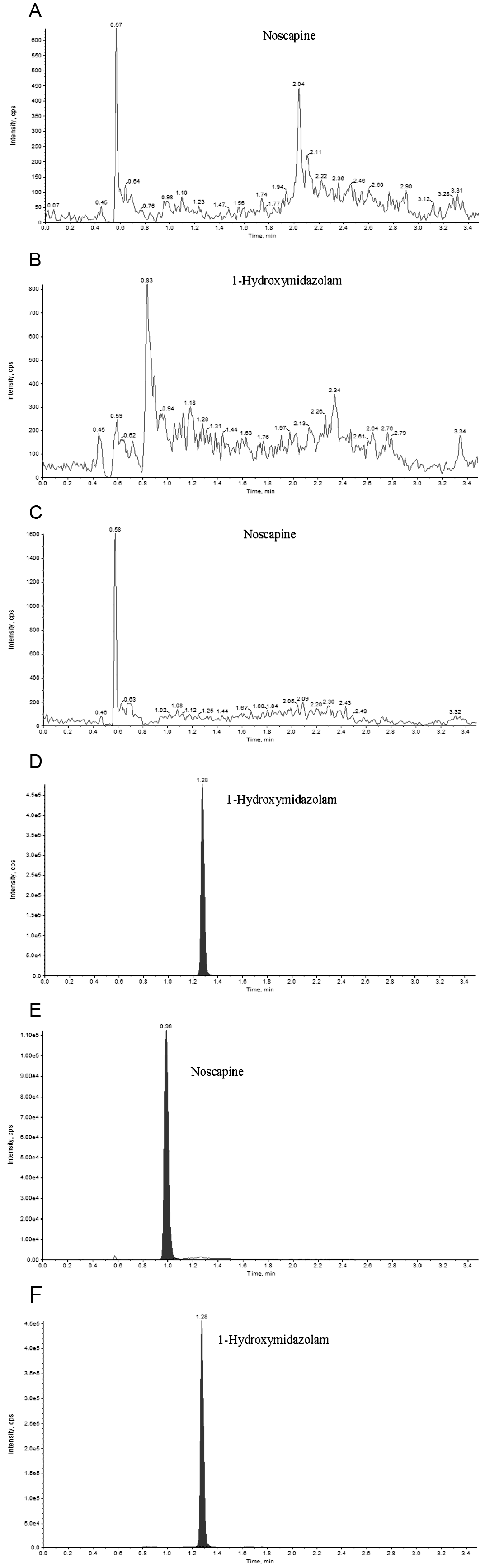

Supplement: Supplementary Figure 2 — Chromatograms of noscapine treated mouse plasma. Chromatograms of noscapine and 1-Hydroxymidazolam measured in (A, B) blank plasma, (C, D) plasma spiked with 1-Hydroxymidazolam (internal standard, 1µg/ml) and (E, F) plasma spiked with noscapine (150ng/ml) and 1-Hydroxymidazolam (internal standard, 1µg/ml). [file Image_2.tif]

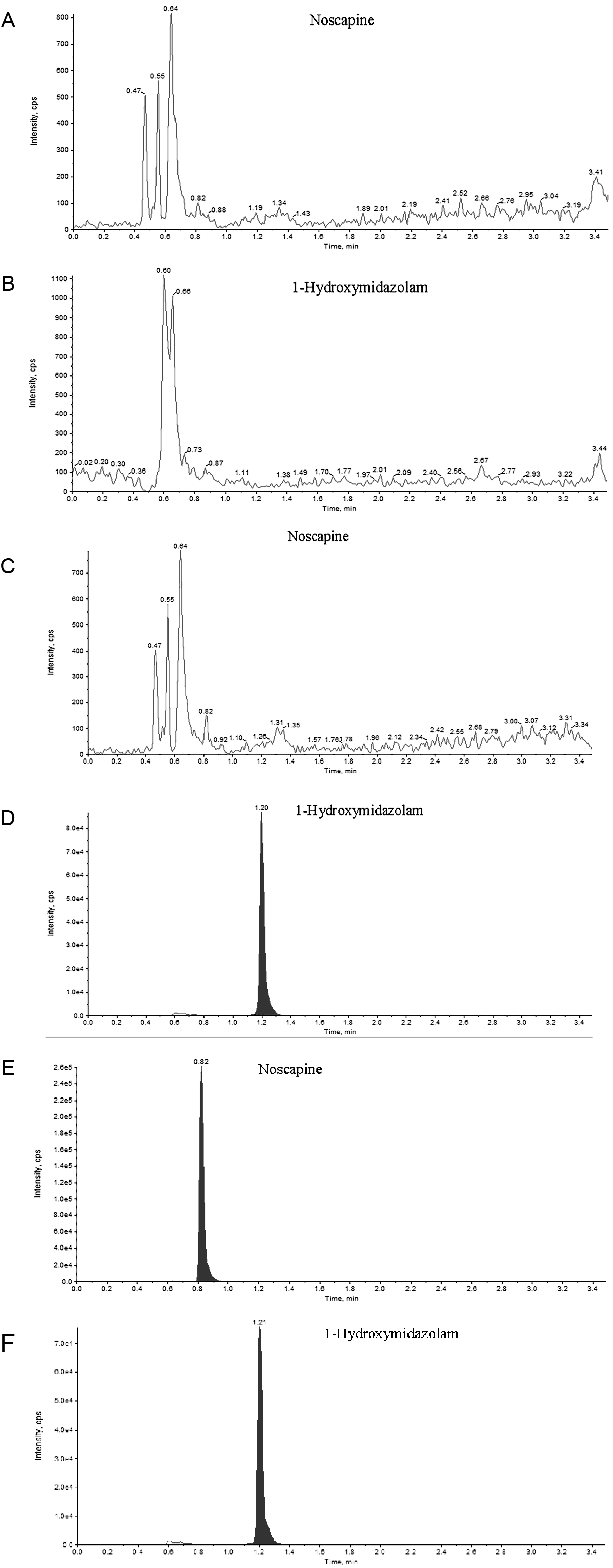

Supplement: Supplementary Figure 3 — Chromatograms of noscapine treated mouse brain homogenate. Chromatograms of noscapine and 1-Hydroxymidazolam measured in (A, B) blank brain homogenate, (C, D) brain homogenate spiked with 1-Hydroxymidazolam (internal standard, 1µg/ml) and (E, F) brain homogenate spiked with noscapine (150ng/ml) and 1-Hydroxymidazolam (internal standard, 1µg/ml). [file Image_3.tif]
